# Acute Care for Elders (ACE) Team Model of Care: A Clinical Overview

**DOI:** 10.3390/geriatrics3030050

**Published:** 2018-08-06

**Authors:** Kellie L. Flood, Katrina Booth, Jasmine Vickers, Emily Simmons, David H. James, Shari Biswal, Jill Deaver, Marjorie Lee White, Ella H. Bowman

**Affiliations:** 1 Division of Gerontology, Geriatrics, and Palliative Care, University of Alabama, Birmingham, AL 35294, USA; kjulian@uabmc.edu (K.B.); jkvicker@uab.edu (J.V.); ehbowman@uabmc.edu (E.H.B.); 2 University of Alabama at Birmingham Hospital, Birmingham, AL 35294, USA; esimmons@uabmc.edu (E.S.); djames@uabmc.edu (D.H.J.); sbiswal@uabmc.edu (S.B.); mlwhite@uab.edu (M.L.W.); 3 Birmingham Veterans Affairs Medical Center, Birmingham, AL 35294, USA; 4 Lister Hill Library of the Health Sciences, UAB Libraries, University of Alabama, Birmingham, AL 35294, USA; jilld@uab.edu; 5 Division of Pediatric Emergency Medicine, University of Alabama, Birmingham, AL 35294, USA

**Keywords:** Acute Care for Elders, ACE Unit, interprofessional team, interdisciplinary

## Abstract

The Institute of Medicine (IOM) Reports of *To Err is Human* and *Crossing the Quality Chasm* have called for more interprofessional and coordinated hospital care. For over 20 years, Acute Care for Elders (ACE) Units and models of care that disseminate ACE principles have demonstrated outcomes in-line with the IOM goals. The objective of this overview is to provide a concise summary of studies that describe outcomes of ACE models of care published in 1995 or later. Twenty-two studies met the inclusion. Of these, 19 studies were from ACE Units and three were evaluations of ACE Services, or teams that cared for patients on more than one hospital unit. Outcomes from these studies included increased adherence to evidence-based geriatric care processes, improved patient functional status at time of hospital discharge, and reductions in length of stay and costs in patients admitted to ACE models compared to usual care. These outcomes represent value-based care. As interprofessional team models are adopted, training in successful team functioning will also be needed.

## 1. Introduction

The launching point for the quality movement in healthcare was the 1999 Institute of Medicine (IOM) Report *To Err is Human,* which concluded tens of thousands of Americans die annually from preventable errors in care [1]. In 2001, the follow-up report *Crossing the Quality Chasm* further described the gap between evidence-based healthcare and care delivered. This 2001 report acknowledged an aging population and thus, an increasing proportion of patients with multiple chronic conditions. The report also called for fundamental redesign of care delivery to include systems to train and support high performing patient-centered teams [2].

Forward thinking geriatric clinicians recognized the need for interprofessional team models of acute care prior to these IOM reports. The Acute Care for Elders (ACE) Unit is one of the better recognized examples. The ACE Unit model consists of several core components: (1) patient-centered care with proactive geriatric assessments, (2) nurse-driven care plans for the prevention and management of geriatric syndromes, (3) comprehensive care transition planning beginning at admission, and (4) medical care review with the goal to prevent iatrogenesis and incident geriatric syndromes. This care is delivered by an interprofessional team that conducts frequent (usually Monday-Friday) team meetings (ACE rounds) to develop the geriatric care plans for each patient. In most ACE Units a geriatrician or geriatric advanced nurse practitioner either participates in the team rounds and/or is an attending practitioner for patients. In addition, ideally the physical environment of the ACE Unit is modified to promote safe mobility and cognitive stimulation [3,4].

The first randomized controlled trial (RCT) of an ACE Unit was published in 1995 by Landefeld et al., and demonstrated the benefits of this care delivery redesign [4]. This publication was a seminal moment, launching ACE Unit development and research that continues today. With this in mind, we present here an overview of studies evaluating outcomes from ACE models and published in 1995 or later. We provide this overview with two primary objectives: (1) to provide a concise reference tool for use by clinicians and researchers working in the field of ACE team models of care, and (2) to assist in identifying opportunities for future ACE efforts, including scaling and dissemination strategies and future quality and research efforts.

## 2. Methods

**Figure 1:**
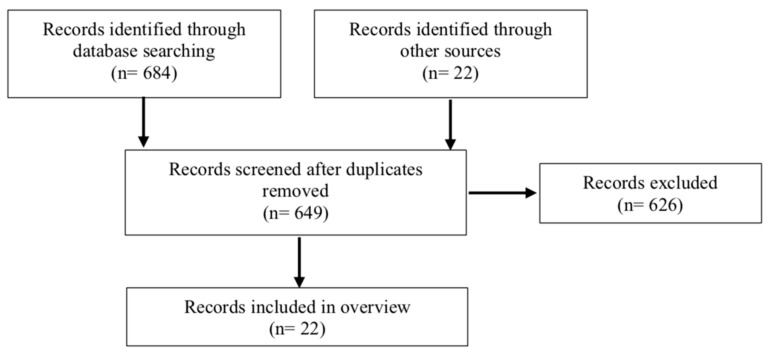
Literature review flow diagram.

Utilizing published criteria for fourteen recognized types of literature reviews, we present here a literature “overview,” defined as “any summary of the medical literature that attempts to survey the literature and describe its characteristics.” Overviews “can provide a broad and often comprehensive summation of a topic area” and the analysis may be “chronological, conceptual, or thematic” [5]. To complete this overview, we conducted a literature search using Pubmed, Embase, CINAHL, and Scopus with the following database-specific subject headings, key terms, and phrases: “Acute Care”; “Acute Care for Elders”; “Interprofessional Teams”; “Interdisciplinary Teams”; “Interprofessional Relations”; “Geriatrics”; “Aged”; “Aged 80 and older”; and “Elderly”. After removing duplicates, two authors (KLF, KB) reviewed all titles and abstracts for studies satisfying our inclusion/exclusion criteria to ensure adherence to the ACE model (Table 1). This was followed by critical reading by four authors (KLF, KB, JV, DJ) of full text articles if insufficient information was provided in the title or abstract to determine relevance. Additionally, the reference lists of included retrieved systematic reviews and meta-analyses were also examined for other articles that would meet the inclusion criteria for this overview (Figure 1). In our final list of relevant articles, we did include one study from an ACE Unit accepting patients with a younger age threshold than traditional ACE units (60 years and over) [6].

## 3. Results

Our search strategy yielded twenty-two articles included in this overview. We present here the key findings from these studies in two categories of ACE models: (1) ACE Units, in which the care occurs for patients admitted to one geographically distinct hospital unit (19 studies), and (2) ACE Services, in which the team care is delivered by an admitting service caring for patients on more than one hospital unit (3 studies). Table 2 and Table 3 are summaries of the key study characteristics and outcomes included in this overview. 

### 3.1. Studies from ACE Units: Geriatric Processes of Care

Of the 19 studies examining ACE Units, five examine geriatric care processes and/or recognition of geriatric syndromes as outcome measures; two are RCTs [9,11,16,17,23]. Both RCTs found significantly increased documentation of geriatric syndromes in patients admitted to ACE compared to UC units [9,11]. In the Counsell et al., RCT, ACE demonstrated significant increased use of nurse-driven geriatric care protocols and earlier and more frequent consultations to social work and physical therapy.

Two non-randomized studies examined care processes in ACE models that did not require the in-person presence of a geriatric specialist [16,23]. Dr. Michael Malone and his team from the Aurora Health Care System have developed ACE Tracker software for use in several electronic medical record (EMR) systems. The ACE Tracker program summarizes in one report geriatric data items documented by various ACE team members (i.e., history of cognitive impairment, results of geriatric screens, presence of potentially inappropriate medications, consultation to social services or rehabilitation therapies). This unit-based ACE Tracker Report is then utilized by an “e-Geriatrician” who can participate in a unit’s ACE rounds via conference call using the same electronic ACE Tracker data. In a pre/post study examining geriatric care processes, the e-Geriatrician model demonstrated significant reduction in the use of urinary catheters and increase in consultations for physical therapists on a surgical and medical unit, but no significant change in other processes measured (use of physical restraints, social service assessments, high risk medications) [16].

In a 2018 pilot study, Booth et al., evaluated the impact of a “Virtual ACE” unit intervention that also does not require geriatrician presence either in person or remotely. Team members from all disciplines on a hospital unit are trained in ACE principles, including use of care protocols stemming from standardized nurse assessments for cognitive impairment, delirium, function, and mobility. ACE Tracker capability also exists in this hospital and use of this tool for recognition of geriatric syndromes and interprofessional team communication was included in the training. In pre/post analysis Booth et al., report a significantly increased proportion of patients receiving geriatric screens for function and delirium on two orthopedic surgery units, signaling it may be possible to embed ACE care processes into the routine workflow of non-ACE units throughout a hospital [23].

### 3.2. Studies from ACE Units: Function and Mobility Outcomes

Likely because one of the primary goals of ACE Units was to prevent hospital acquired disability, eleven of the ACE Unit studies evaluated patient performance of activities of daily living (ADL), instrumental activities of daily living (IADL), mobility, and/or discharge to a post-acute care facility, either separately or as part of composite outcome measure [4,6,7,9,10,12,13,15,18,21,23]. Six of these studies were randomized and one was a secondary analysis of data collected from a prior RCT [4,7,9,10,12,18,21]. The landmark Landefeld et al., study randomized general medical patients age 70 and over to ACE and UC units at a teaching hospital and evaluated function as the primary outcome. Significantly more ACE patients improved performance of ADLs from baseline and admission to time of discharge compared to UC [4]. ACE patients also experienced significantly less post-acute care facility placement (14% vs. 22 %, *p* = 0.01). A second RCT from this same investigator group evaluated a new ACE Unit in a community hospital [9]. This study did not demonstrate the same benefits in ADL performance but did demonstrate significant improvement in the composite outcome of ADL decline or need for post-acute care facility placement. The authors noted the logistical challenges in the non-teaching environment due to lack of a physician representative present to participate in the ACE rounds. A third RCT by Barnes et al., also did not demonstrate improvements in function or post-acute care facility referral [18]. Of note, this RCT was conducted on the same ACE unit as in the Landefeld study and the authors noted some of the ACE care processes and environmental redesign were disseminated to UC units during the study period. Finally, a RCT from an ACE Unit in Sweden evaluated a composite outcome measure of death and/or severe ADL dependence and/or poor psychological well-being 3 months after discharge and found no significant difference in ACE versus UC [10]. Another non-USA randomized study of an ACE model for hip fracture patients also did not find a significant difference in a composite outcome that included mobility measures 6 months after surgery, except in subgroup analysis of cognitively impaired patients [12].

Several non-randomized studies did report significantly improved ADL performance at time of hospital discharge and/or reductions in post-acute care facility use. Two of these studies came from non-USA-based ACE Units that utilized geriatricians as the attendings [6,15] and one from implementing ACE care principles on a stroke unit [13]. In addition, the previously described Virtual ACE model reported increased patient mobilization [23]. In a study utilizing hospital units as the target for randomization, Borenstein et al. describe a unit-based care redesign intervention similar to the Virtual ACE approach but also included team communication notes with decision support in the EMR. The intervention units were also trained to conduct daily interprofessional team huddles facilitated by an in-person ACE physician advisor who was not directly caring for the patients. Analysis of data, which included patients who screened positive for defined geriatric risk factors, found medical complications were reduced and ACE patients had a significantly increased odds ratio of discharge to a facility [21]. Similar to Barnes et al., the authors noted that ACE care plans were also present to some extent in the comparison units [21].

### 3.3. Studies from ACE Units: Additional Outcomes

Several studies demonstrated other patient outcomes including but not limited to significantly improved self-rated overall health status [4] and health related quality of life [22]; improved patient, caregiver, and provider satisfaction [9]; and reduced complications of care [21]. One study, a RCT targeting older frail patients, evaluated mortality as the primary outcome and reported significantly reduced mortality at 3 and 6 months post-discharge from ACE compared to a UC unit [11]. A more recent study also targeted older frail patients and also noted reduced 3-month mortality [22].

### 3.4. Studies from ACE Units: Healthcare Utilization

In addition to the use of post-acute care facilities as summarized above, fifteen of the unit-based studies reported healthcare utilization (length of stay, readmissions, costs) either as primary or secondary outcome [6,7,8,10,11,12,13,14,15,16,18,19,20,21,22]. Ten of these demonstrated either significant reduction or trends toward reduced length of stay (LOS) and/or costs. More recent studies signal the ACE model may reduce costs even in the setting of short LOS. In a 2013 retrospective cohort study of hospitalist patients age 70 and over admitted to ACE versus UC hospitalist units, variable direct costs were significantly reduced despite the fact that both units had a LOS less than 4.3 days [20]. None of the retrieved studies evaluated 30-day readmissions as a primary outcome. Those that included readmissions as a secondary outcome reported either reduced or unchanged readmission rates even when LOS was reduced with ACE team care.

### 3.5. Studies from ACE Services

The literature search yielded three publications describing service-based ACE models (Table 3). In these models, patients admitted to one of these services received ACE-like care even if they were located on different units. The Mobile-ACE (MACE) study specifically states one of the aims is to deliver geriatric care “without the limitations of a physical unit.” This model consisted of a geriatrician as the attending with a geriatric fellow, social worker, and clinical nurse specialist assigned to the service rather than a hospital unit. The MACE team conducted daily team meetings addressing geriatric care needs and early care transition planning. This model resulted in significantly reduced adverse events and LOS and an increase in care transitions preparedness as measured by the 3-item the Care Transitions Measure [26,27].

The remaining ACE service studies both utilized an intensive geriatric education experience delivered to general medical teams coupled with implementation of ACE care processes (geriatric screens, care protocols, and interprofessional team meetings). In Wald et al., this approach was implemented with Hospitalists to create the Hospitalist-ACE Service that admitted patients preferentially to 12 designated beds but also applied the ACE care and studied the impact in patients on any unit. The team conducted daily (Monday-Friday) 15-min team huddles to coordinate geriatric and care transition plans. This model demonstrated significantly increased recognition of geriatric syndromes and utilization of Do Not Resuscitate orders but did not demonstrate changes in LOS or costs in this program’s first year in operation [24]. Yoo et al., implemented the same approach for general medicine services at an academic medical center. The intervention included daily geriatric assessments for function, cognition, delirium, medication reconciliation, and sleep disturbance and three times weekly 45-min interprofessional team meetings. The per-protocol analysis, which only included patients who received at least 80% of the daily geriatric screens, demonstrated reduced LOS (6.1 days vs. 6.8 days, *p* = 0.008) [25].

## 4. Discussion

Our review of the twenty-two studies retrieved revealed several themes. The majority of studies are not randomized trials, and those that are occurred predominantly in the first decade of ACE Unit research. One possibility for this limited number of RCTs may be the logistical inability to “hold beds” on designated hospital units due to continually high patient census levels. Non-randomized studies have the associated limitations and therefore any findings and conclusions should be considered within this context. Service related studies are difficult to perform, leading to the disparate study types our search revealed. The lack of standardized and uniform outcome measures or metrics for ACE Units further limits the ability to compare the effectiveness of the various ACE interventions. Many of the ACE studies we found were implementation or feasibility studies and fit into the category of pragmatic trials. Glasgow et al., summarizes the impetus behind the call for increasing the number of pragmatic studies, defined as trials “designed to answer the question of whether a program works under usual conditions, compared to explanatory trials that answer the question if an intervention works under ideal conditions” [28].

We also observed the methodology by which ACE care is implemented has begun to expand. Most commonly, ACE Units continue to care for general medical patients with a unit-based interprofessional team and in-person involvement from a geriatric trained clinician. However, likely in part due to the improved outcomes demonstrated by ACE studies and the increasing numbers of geriatric patients on all units, ACE models are increasingly being deployed for subspecialty populations and operationalized on units and services with and without direct geriatrician participation. We included these studies of ACE care in subspecialty patient populations (stroke, orthopedic surgery) not necessarily for direct comparison to studies of general medical patients, but rather to reveal the dissemination and possible benefits of the ACE model for older adults throughout a hospital.

The mixed results observed in some of the outcomes across studies may be related to the ACE intervention implemented, study design limitations, the outcome measurement tools, or other factors. For example, several studies evaluated outcomes within the first year of operation of a new ACE program. Given the time required to develop high-performing teams in systems accustomed to functioning in silos, repeat evaluation of these models may be beneficial now that time has elapsed. The two studies that demonstrated longer LOS from ACE were both from non-USA hospitals over 15 years ago. Overall the ACE studies in which the usual care LOS was six or more days demonstrated LOS reductions. Cost savings can most easily be attained by reducing LOS. However, ACE Units have also demonstrated reduced costs of care even when LOS is short. This perhaps signals the role of ACE in delivering care that is aligned with patient goals, therefore avoiding unnecessary interventions. Some ACE studies reported increased formal consultation to allied health services such as nutrition and rehabilitation therapies. Another possible future outcome for study may be appropriateness of these referrals, given these resources are often limited and the mandate for stewardship of resources in today’s healthcare system. The mixed results in the post-discharge function outcomes measured may signal another opportunity for future intervention and investigation. Additionally, given the barriers of saving beds discussed above, we suspect many ACE Units today admit geriatric patients with varied potential for clinical and functional improvement. As one example, the University of Alabama at Birmingham ACE Unit does not utilize any ACE specific admission criteria. This has resulted in an ACE team caring for geriatric patients ranging from fully independent in ADLs to those receiving end-of-life care. Thus, the primary focus of this ACE Unit has become the delivery of “goal-aligned care”. This heterogeneous mix of patients with disparate goals will likely require alternative research approaches and measurement tools.

Finally, a common theme in the ACE literature is the prerequisite for training healthcare professionals in the basic principles of geriatric medicine. Given the national shortage of fellowship trained geriatricians, attention to equipping all healthcare providers with this knowledge is essential to disseminating ACE care and achieving the ultimate goal to establish Age-Friendly Health Systems [29]. National institutions and funding mechanisms are required to support this education. Examples of such support include the Nurses Improving Care for Healthsystem Elders institution [30] and the Health Resources and Services Administration funded Geriatric Workforce Enhancement Programs [31]. The basic training of all healthcare providers in geriatrics could then shift the role of fellowship trained geriatricians to lead more system-level initiatives to further promote age-friendly care delivery [32]. Extending beyond geriatric education, working in teams has been recognized as a mechanism for improving quality and care coordination for patients of all ages. Future opportunities may also include merging patient safety teamwork curriculum such as TeamSTEPPS^®^ (Team Strategies & Tools to Enhance Performance & Patient Safety) with ACE team training. Available from the Agency for Healthcare Research and Quality [33], TeamSTEPPS^®^ use has resulted in sustained improvements in patient safety [34]. In addition to the challenges of providing geriatric education to all healthcare providers, other barriers hospitals face in developing ACE Units include lack of hospital-based geriatric providers with protected time and effort to lead program development, challenges in securing dedicated unit space, and the need for some start-up funding, despite published evidence of return on this investment. While dedicated ACE Units will likely remain the core model, the future for ACE care, and a means to address development barriers, may reside in the current trend toward embedding ACE care processes into existing hospital systems and workflows.

## 5. Conclusions

The patient characteristics and outcome measures in these 22 ACE studies are varied. While we have summarized key variables that may influence outcomes (i.e., mean age, mortality rates), conducting an in-depth systematic review and meta-analysis is beyond the scope of this literature overview. Rather, we conducted this overview with the goal to concisely summarize ACE studies to serve as a reference tool for clinicians and researchers working to improve and expand the implementation of team care for all hospitalized elders. Despite our attempt to be thorough in our search we suspect there are additional ACE unit studies that were not retrieved with our strategy. We also did not conduct an in-depth rating of the quality of each study retrieved. Overall, the majority of the studies demonstrated improved quality with simultaneous reductions in measures of inefficiency (LOS) that were cost-neutral or cost-saving. These outcomes represent value-based care and are aligned with the goals the IOM called for almost two decades ago.

## Figures and Tables

**Table 1:** Study inclusion and exclusion criteria.

Study Characteristics	Inclusion Criteria	Exclusion Criteria
Type	Randomized controlled, prospective or retrospective cohort, observational, case-control, implementation, feasibility, or quality improvement full-text studies with an intervention and comparator group	Descriptive studies of the ACE unit model or patient population without a comparator group Abstracts, dissertations, and book chapters
Population	Patients aged ≥ 65 years	Non-geriatric patient population
Intervention	Contains core components of ACE model (geriatric assessment and management by an interprofessional team consisting of at least three different healthcare professionals targeting comprehensive geriatric care) Intervention delivered on a hospital unit to which patient was admitted for an acute illness	Geriatric consult or co-management models of care Intervention included an outpatient component (i.e., home evaluation, follow-up in a geriatric clinic) Intervention designed to target only one diagnosis (i.e., delirium) and not comprehensive geriatric care Intervention delivered in a non-acute care unit setting (i.e., emergency department) or on a unit to which a patient was transferred for a rehabilitation focus after stabilization of an acute illness
Comparator Group	Usual/routine hospital care in a similar patient population on the same or similar type of unit or service	No comparator or usual/outline care group
Outcome Measures	Patient related geriatric care process, clinical, satisfaction, quality of life, or healthcare utilization outcomes	Staff or provider educational or perception outcomes
Language	English	Non-English
Publication date	1995 or later	Prior to 1995

ACE = Acute Care for Elders.

**Table 2:** ACE unit studies and key findings *.

Study (Setting)	Design	Patient Population (Mean Age of ACE Cohort)	ACE vs. UC Attending Physicians (Sample Sizes)	Primary Outcome Measured (ACE vs. UC)	Secondary Outcome(s) Measured (ACE vs. UC)	Study and/or Intervention Limitations	Study Strengths and/or Intervention Innovations
Landefeld et al., 1995 [4] (university hospital, USA)	RCT	General medical patients aged ≥ 70 years (80.2)	Internists on ACE (327) and UC (324) units	Significantly improved ADL performance from baseline (*p* = 0.05) and admission (*p* = 0.009) to discharge	Significantly reduced PACF placement (14% vs. 22 %, *p* = 0.01) Significantly improved overall health status (*p* < 0.001) at discharge No difference in hospital charges	Utilized charges for cost analysis	Randomized Improved outcomes in subgroup and multivariate analyses
Covinsky et al., 1997 [7] (university hospital, USA)	RCT; Cost analysis from Landefeld et al., study	General medical patients aged ≥ 70 years (80.7)	Internists on ACE (326) and UC (324) units	No significant reduction in total costs per case ($6608 vs. $7240, *p* = 0.93)	No significant reduction in LOS (7.5 vs. 8.4 days, *p* = 0.449) Significantly reduced 90-day PACF use (24.1% vs. 32.3%, *p* = 0.034)	Total costs includes indirect costs Lacked power to determine significance in cost difference	Randomized Included ACE start-up costs, likely under-estimating long-term cost savings
Stewart et al., 1999 [8] (community teaching hospital, USA)	Prospective observational	General medical or surgical patients aged ≥ 75 years (86)	Internist or Surgeon on ACE (34) and UC (27) units	No significant difference in LOS (6.0 vs. 7.1 days, *p* = 0.06)	Significantly reduced charges ($6,223 vs. $10,042, *p* < 0.01)	Non-randomized Multiple significantly different baseline characteristics between cohorts and results unadjusted Utilized charges for cost analysis	ACE care for medical and surgical patients
Counsell et al., 2000 [9] (community teaching hospital, USA)	RCT	Community-dwelling general medical patients with LOS ≥ 2 days aged ≥ 70 years (80)	Internist or Family Practice attending on ACE (767) and UC (764) units	No significant difference in ADL performance at discharge Significant reduction in composite outcome (ADL decline or PACF placement; 34% vs. 40%, *p* = 0.027)	Significant increased use of nursing care plans (79% vs. 50%; *p* = 0.001), SW consults (50% vs. 43%, *p* = 0.012), and PT consults (42% vs. 36%, *p* = 0.027) Significant reduction in restraint use (2% vs. 6%; *p* = 0.001) Improved patient, caregiver, and provider satisfaction	ADL outcome may have been influenced by healthier patient population and shorter LOS than Landefeld et al., RCT	Randomized Large sample size Studied ACE in patients not on a teaching service
Asplund et al., 2000 [10] (university hospital, Sweden)	RCT	General medical patients aged ≥ 70 years (80.9)	Internist initially followed by Geriatrician on ACE (190) vs. Internist on UC (223) units	No significant difference in poor global outcome measure ^α^ 3 months post-discharge (RR 1.06; 95% CI 0.84–1.34)	Significantly reduced LOS (5.9 vs. 7.3 days, *p* = 0.002) No difference in readmissions or healthcare utilization at 3 months No significant difference in hospital mortality (4% vs. 3%)	Per-protocol analysis performed since majority of ineligible patients were due to inappropriate randomization processes	Randomized Evaluated function and well-being post-discharge
Salvedt et al., 2002 [11] (university hospital, Norway)	RCT	General medical patients meeting frailty criteria aged ≥ 75 years (81.8)	Geriatrician on ACE (127) vs. Internist or Medical Subspecialists on UC (127) units	Significantly reduced mortality at 3 (12% vs. 27%, *p* = 0.004) and 6 months (16% vs. 29%, *p* = 0.02) post-discharge	Significantly increased LOS (15 vs. 7 days, *p* < 0.001) Significantly more ACE patients with dementia, depression, and delirium diagnoses documented (38% vs. 7%, *p* < 0.001)	Non-USA based study may have influenced LOS Some ACE patients were transferred from other units	Randomized Targeted frail patients First to evaluated mortality as a primary outcome
Naglie et al., 2002 [12] (university hospital, Canada)	RCT	Patients with surgical hip fracture repair aged ≥ 70 years (83.8)	Geriatrician directed medical care on Ortho-ACE (141) vs. Internist directed medical care on Ortho-UC (138) units	No significant difference in composite outcome (% patients alive with no decline from baseline in ambulation, transfers, or place of residence 6 months post-surgery; adjusted OR 1.1, 95% CI 0.6–2.1)	Significant increase in % patients with composite outcome (alive with no decline from baseline in ambulation, transfers, or place of residence at 6 months) in analysis of cognitively impaired patients (47% vs. 24%, *p* = 0.03) Significantly increased LOS (29.2 vs. 20.9 days, *p* < 0.001)	Non-USA based study may have influenced LOS Did not evaluate in-hospital outcomes	Randomized design Extension of ACE to orthopedic surgery patients Twice weekly ACE rounds
Allen et al., 2003 [13] (community teaching hospital, USA)	Pre/post comparison	Acute stroke patients pre/post launch of ACE-like stroke unit; no age criteria reported (72)	Neurologist 1-year pre (622) vs. 1-year post (544) stroke unit utilizing ACE model	Significantly reduced LOS (3.8 vs. 4.6 days, *p* < 0.0001)	Significantly more patients discharged home (62% vs. 50%, *p* < 0.0001) Significantly increased proportion of patients without a readmission at 1-year (41% vs. 18%, *p* < 0.0001) Significantly reduced health system Medicare stroke-specific and risk-adjusted inpatient mortality (11.4% vs. 8.4%, *p* = 0.02) No significant difference in all-cause mortality (7% vs. 16%; *p* = 0.11)	Non-randomized Data from administrative database	ACE team model and processes used to develop an acute stroke unit
Jayadevappa et al., 2006 [14] (university hospital, USA)	Retrospective case-control	General medical patients admitted for CHF, UTI, or pneumonia aged ≥ 65 years (79.6)	Internist or Geriatrician on ACE (680) vs. Internist on UC (680) units	Significantly reduced LOS (4.9 vs. 5.9, *p* = 0.01) Significantly reduced mean costs ($13,586 vs. $15,039, *p* = 0.012)	Reduced annual readmissions after controlling for age, race, comorbidities, and number of prior admissions	Non-randomized Data from administrative database Costs estimated from a cost-to-charge ratio	Adjusted for prior admissions in analyzing readmission rate
Zelada et al., 2009 [15] (military teaching hospital, Peru)	Prospective observational	General medical patients aged ≥ 65 years (79.6)	Geriatrician on ACE (68) vs. Internist on UC (75) units	Significantly reduced ADL decline during hospitalization (19% vs. 40%, *p* = 0.013)	Increased OR for ADL decline in UC patients (4.24; 95% CI 1.50–11.9) Reduced LOS on ACE (7.5 vs. 9.92 days, *p* = 0.03)	Non-randomized Multiple significantly different baseline characteristics between cohorts	Once weekly ACE rounds
Malone et al., 2010 [16] (community hospital, USA)	Pre/post comparison	General medical or urology patients aged ≥ 65 years (no mean age reported)	Urology or Internist on medical-surgical units pre (478) vs. post (406) e-Geriatrician	Significantly reduced use of urinary catheters (26.2 vs. 20.1%, *p* = 0.03) Significantly increased physical therapy referrals (27.0% vs. 39.1%; *p* < 0.001)	No significant difference in use of physical restraints, social service assessments, high-risk medications, LOS, or 30-day readmissions	Non-randomized No formal tracking of whether recommendations made by geriatricians are followed	Use of EMR tool to disseminate ACE care Twice weekly e-Geriatrician in ACE rounds
Flood et al., 2011 [17] (university hospital, USA)	Retrospective chart review	Hematology-oncology patients with nutritional deficits aged ≥ 65 years (75.25)	Private Oncologist or Teaching attending with residents on Oncology-ACE (103) vs. UC (82) units	Significantly increased OR for receiving a formal nutrition consult (2.1, 95% CI 1.033–4.300) and nutritional supplements ordered (2.5; 95% CI 1.221–5.319) in adjusted analysis	Significantly increased proportion of patients receiving a nutrition consult (63.1% vs. 45.1%, *p* = 0.011) and an order for supplements (57.3% vs. 32.9%, *p* = 0.001) in unadjusted analysis	Non-randomized No standardized nutritional risk screening process on units Significantly more OACE cohort with low BMI No clinical outcomes measured	Extension of ACE model to hematology/oncology patients Evaluated role of ACE on nutritional processes of care
Barnes et al., 2012 [18] (university hospital, USA)	RCT (2nd RCT from same ACE unit in Landefeld et al., study)	Community-dwelling general medical patients aged ≥ 70 years (81)	Internists on ACE (858) and UC (774) units	Significantly reduced LOS (6.7 vs. 7.3 days, *p* = 0.004) Significantly reduced cost per patient ($9,477 vs. $10,451, *p* < 0.001)	No significant difference in ADL, IADL, or mobility performance at discharge	Gap between time study conducted (1993–1997) and publication (2012)	Randomized Large sample size
Ahmed, et al., 2012 [19] (university hospital, USA)	Pre/post comparison	General medical patients aged ≥ 70 years (no mean age reported)	Geriatrician or Geriatric Consultant with Private Internist post (1096) vs. Private and Teaching Internist on UC (383) 1-year pre-ACE	Significant reduction in LOS (5.55 vs. 7.76 days; *p* = 0.001) and CMI adjusted LOS (5.16 vs. 6.40; *p* = 0.007) year 2 vs. baseline	No significant difference in direct costs Reduced readmission rate from baseline to years 1 and 2 combined (14.04% vs. 11.95%; no statistical analysis performed)	Non-randomized Baseline patients from multiple different units Unequal sample sizes and time periods in pre- vs. post-cohorts	Measured CMI adjusted LOS Measured direct costs
Perez-Zepeda et al., 2012 [6] (community hospitals, Mexico)	Prospective matched cohort	General medical patients with ≥ 1 targeted geriatric syndrome aged ≥ 60 years (72.6)	Geriatricians on ACE (70) vs. Internist on UC (140) units	Significantly lower adjusted OR of composite outcome (presence of ADL decline, pressure ulcer, delirium, or death; 0.27; 95% CI 0.10–0.70)	Significantly reduced OR for ADL decline (0.23; 95% CI 0.08–0.65) No significant difference in LOS (9.9 vs. 9.3 days, *p* = NS) No significant difference in adjusted OR for hospital mortality (1.50; 95% CI 0.31–7.18)	Non-randomized Small sample sizes for two-year study recruitment period	Targeted patients with existing geriatric syndromes
Flood et al., 2013 [20] (university hospital, USA)	Retrospective cohort	General medical patients aged ≥ 70 years (81.6)	Hospitalists on ACE (428) and UC (390) units	Significantly reduced variable direct costs ($2109 vs. $2480, *p* = 0.009)	Significantly reduced 30-day readmissions (7.9% vs. 12.8%, *p* = 0.02) No significant difference in discharge destination (*p* = 0.12) including death in hospital 1.4% vs. 1.8%)	Non-randomized Data from administrative database	Units had same attendings Measured variable direct costs Reduced costs despite short LOS
Borenstein, et al., 2016 [21] (university hospital, USA)	Cluster RCT of hospital units	General medical patients with geriatric risk factors aged ≥ 65 years (81.1)	Internist on medical units with (792) and without (592) ACE training and workflow redesign	Observed:Expected LOS ratio < 1 with ACE intervention and >1 on UC	Significantly reduced adjusted OR of any complication (0.45, 95% CI 0.21–0.98; *p* = 0.043) or transfer to ICU (0.45; 95% CI 0.25–0.79; *p* = 0.006) Significantly increased adjusted OR of discharge to PACF (1.43, 95% CI 1.06-1.93; *p* = 0.021) No significant difference in adjusted OR of hospital mortality (0.69, 95% CI 0.42–1.15; *p* = 0.16)	Non-randomized Amount of uptake of NICHE care protocols on control units unknown	Large sample size Redesigned workflows of all unit personnel to include ACE care processes
Ekerstad et al., 2017 [22] (community hospital, Sweden)	Prospective controlled	General medical patients with positive frailty screen aged ≥ 75 years (85.7)	Internist, Family Practitioner, and/or Geriatricians on ACE (206) vs. Internist on UC (202) units	Significantly reduced adjusted OR of decline in HRQOL (vision, ambulation, dexterity, emotion, cognition, pain dimensions) 3 months post-discharge	Significantly reduced 30-day readmission (19% vs. 28%, *p* = 0.048) Reduced adjusted 3-month mortality (HR 0.55, 95% CI 0.32–0.96) No significant difference in hospital mortality (4% vs. 5%, *p* = 0.6)	Reports trial is randomized but patients assigned to ACE or UC based on bed availability	Targeted older frail patients Evaluated quality of life
Booth et al., 2018 [23] (university hospital, USA)	Pre/post comparison	Orthopedic surgery or medical patients aged ≥ 65 years (74.4)	Orthopedic Surgeon or Hospitalist pre (48) vs. post (113) ACE workflow redesign	Significantly improved completion of geriatric screens for ADL (62.5% vs. 88.5%, *p* < 0.001) and delirium (4.2% vs. 96.5%, *p* < 0.001)	Significantly increased patients mobilized bed to chair (36.4% vs. 63.5%, *p* < 0.05) No significant difference in patients ambulating in hallway or delirium prevalence	Non-randomized Small and unequal sample sizes/time periods in cohorts limits ability to measure significance	Extension of ACE to orthopedic surgery patients Extension of ACE without geriatric specialist

RCT = randomized controlled trial; ACE = Acute Care for Elders; UC = usual care; ADL = activities of daily living; PACF = post-acute care facility; SW = social work; PT = physical therapy; RR = relative risk; LOS = length of stay; CHF = congestive heart failure; UTI = urinary tract infection; CI = confidence interval; OR = odds ratio; EMR = electronic medical record; OACE = Oncology-Acute Care for Elders; BMI = body mass index; IADL = instrumental activities of daily living; CMI = case mix index; NICHE = Nurses Improving Care for Healthsystem Elders; HRQOL = health related quality of life; HR = hazard ratio. ^α^ global poor outcome measure = death and/or severe ADL dependence and/or poor psychological well-being. * Table modified and reprinted with permission from Malone M, Capezuti E, Palmer R. (eds) Geriatrics Models of Care: Bringing “Best Practice” to an Aging America, copyright Springer Publishing International Switzerland 2015.

**Table 3:** ACE service studies and key outcomes *.

Study (Setting)	Design	Patient Population (Mean Age of ACE Cohort)	ACE vs. UC Attending Physicians (Sample Sizes)	Primary Outcome Measured (ACE vs. UC)	Secondary Outcome(s) Measured (ACE vs. UC)	Study and/or Intervention Limitations	Study Strengths and/or Intervention Innovations
Wald et al., 2011 [24] (university hospital, USA)	Retro-spective chart review of patients randomized at time of admission	General medical patients aged ≥ 70 years (80.5)	Hospitalist on ACE (122) vs. Hospitalist or General or Subspecialty Internist on UC (95) services	Significantly increased patients with documented recognition and treatment plan for functional (68.9% vs. 35.8%, *p* < 0.0001) and cognitive impairment (55.7% vs. 40%, *p* = 0.02) Significantly increased patients with DNAR orders (39.3% vs. 26.3%, *p* = 0.04)	No significant differences in use of physical restraints, sleep aids, falls, discharge location, LOS, charges, or 30-day readmissions	Hospitalist-ACE attendings rotated on UC services Intervention did not include geriatric training for nurses LOS and 30-day readmission rates low at baseline, limiting ability to improve	30% of ACE patients located off unit and received ACE care Hospitalist-ACE delivered geriatric training to residents Extension of ACE without geriatric specialist
Yoo et al., 2013 [25] (university hospital, USA)	Prospective matched cohort	Community-dwelling general medical patients aged ≥ 65 years (no mean age reported; 43% of patients aged ≥ 80)	Internist on ACE (236) and UC (248) services	No significant difference delirium prevalence (23% vs. 21%, *p* = 0.34)	Significantly reduced LOS (6.1 vs. 6.8 days, *p* = 0.008) No significant difference in 30-day readmissions	Non-randomized Per-protocol and not intention-to-treat analysis UC physicians received geriatric education	Extension of ACE without geriatric specialist
Hung et al., 2013 [26] (university hospital, USA)	Prospective matched cohort	General medical patients aged ≥ 75 years (85.2)	Geriatrician on ACE (173) vs. Internist on UC (173) services	No significant difference in 30-day readmissions (15.4% vs. 22.4%, *p* = 0.21) Significantly fewer patients experiencing an adverse event (CAUTI, restraint use, fall, or pressure ulcer; 9.5% vs. 17.1%, *p* = 0.02) Significantly reduced LOS (4.6 vs. 6.8 days, *p* = 0.001)	Significantly improved 3-item CTM mean score (72.5 vs. 64.9, *p* = 0.01) No significant difference in discharge location, ADL or IADL performance 30 days post-discharge, overall health status, or HCAHPS top box satisfaction scores No significant difference in 30-day mortality (7.5% vs. 5.8%, *p* = 0.51)	Non-randomized ACE service only admitted patients receiving primary care in geriatric patient-centered medical home	Service includes geriatrician attending with allocated social worker and clinical nurse specialist

ACE = Acute Care for Elders; UC = usual care; DNAR = Do Not Attempt Resuscitation; LOS = length of stay; ADL = activities of daily living; IADL = instrumental activities of daily living; CAUTI = catheter associated urinary tract infection; HCAHPS = Hospital Consumer Assessment of Healthcare Providers and Systems; CTM = Care Transition Measure. * Table modified and reprinted with permission from Malone M., Capezuti E., Palmer R. (eds) Geriatrics Models of Care: Bringing “Best Practice” to an Aging America, copyright Springer Publishing International Switzerland 2015.
